# Evaluation of an Online Training for Supervisors of Young Agricultural Workers

**DOI:** 10.3390/ijerph181910395

**Published:** 2021-10-02

**Authors:** Diane S. Rohlman, Megan TePoel, Shelly Campo

**Affiliations:** 1 Occupational and Environmental Health, University of Iowa, Iowa City, IA 52242, USA; megan-tepoel@uiowa.edu; 2 Community and Behavioral Health, University of Iowa, Iowa City, IA 52242, USA; shelly-campo@uiowa.edu

**Keywords:** agriculture, youth, occupational safety, Total Worker Health, health promotion, intervention, health communication

## Abstract

Adolescents and young adults (<25 years) working in agriculture are at greater risk of injury than youth working in other industries. Supervisors play an important role in protecting these young workers who lack workplace experience and whose bodies and brains are still developing. A theoretically based approach was used to develop an online training for supervisors of young agricultural workers. The training addresses an expanded view of occupational safety that not only addresses injury prevention, but also focuses on health promotion and worker well-being using a Total Worker Health approach. A pre-post/post study design was used to evaluate the training. Questionnaires included demographics, workplace characteristics, knowledge, beliefs about protecting young workers, and supervisors’ communication behaviors. One-hundred-eighty-two participants completed all parts of the efficacy trial. A post-test administered immediately after completing the training, indicated that supervisors had greater understanding of the risks to young workers and at 3-month follow-up were more likely to engage in communication behaviors to protect the safety and health of young workers. Positive changes in when, how, and under what circumstances supervisors talk about safety and health occurred. Establishing patterns of protective behaviors in the workplace can have lifelong impact, particularly among young workers.

## 1. Introduction

Youth begin working in agriculture work at younger ages and in more hazardous jobs than youth in other industries [1]. In the United States, eighteen percent of all hired farmworkers are under the age of 25 [2]. Half of all fatal injuries to young workers occur in agriculture [3]. Workplace injury and illness can lead to enduring disabilities. Various factors put young workers at increased risk. There are no federal restrictions on the number of hours children can work on the farm, but there are some state restrictions [1]. Moreover, if the farm is owned and operated by relatives, there are no age or work restrictions of any kind [4]. Younger workers face additional challenges, including lack of previous work experience and training that contribute to the inability to recognize hazards, physical and cognitive limitations based on their stage of development, reluctance to ask questions or to admit that they do not understand something, and they are often assigned more physically demanding tasks [5]. Technology use on the job can also impact safety [6]. Off-the job factors impair focus on the job, including family and school demands and stress, risky behaviors (e.g., substance use), fatigue, and sleep [7,8,9,10,11,12,13,14]. Therefore, it is essential for workplaces to protect the well-being of these vulnerable workers.

Interventions that focus on protection from work-related hazards and address health and well-being are more effective than addressing just one [15,16,17,18,19]. This integration of health protection and health promotion is the foundation of the National Institute for Occupational Safety and Health Total Worker Health™ (TWH) Program (http://www.cdc.gov/niosh/twh, accessed on 31 July 2021) [20]. TWH strategies expand traditional definitions of workplace hazards (e.g., pesticide exposure, unguarded machinery, livestock) to include additional hazards that can also impact health and safety (e.g., stress, fatigue, inexperience). Solutions that impact the workplace (i.e., eliminating hazards, engineering controls, policies, supervisor behaviors) are often more effective and sustainable than those targeting the individual worker (i.e., encouraging health behaviors, use of personal protective equipment (PPE)) [21]. Rather than targeting individual behaviors, the Social Ecological Model (SEM) suggests targeting the environment around the individual, including the interpersonal, organizational, and policy levels of influence [4,22,23]. Young workers in agriculture are influenced by their own knowledge and attitudes, their interactions with supervisors, coworkers, teachers, and workplace policies.

To protect this vulnerable population, an online training using a TWH approach for supervisors of young agricultural workers was developed using several theoretical perspectives to increase its likelihood of efficacy [24]. The goal of this study was to evaluate the training for changes in supervisors’ attitudes, knowledge, and behaviors and the stability of these changes over time. A pre-post/post efficacy trial was used to evaluate the impact of an online supervisor training.

## 2. Materials and Methods

### 2.1. Participant Recruitment

Recognizing the range of settings where young agricultural workers are employed, “supervisor” included employers, parents, and educators. Adults who hire, teach, or train young workers (less than 21 years of age) were invited to participate in the study. A project website was created with information describing the study and contained a link to the study materials. Recruitment began in October 2017 and continued through August 2019. A multipronged approach was used to recruit participants that included conference presentations and recruitment at events, use of an agricultural list-serv, and targeted emails and social media posts through partner organizations and commodity groups.

### 2.2. Online Supervisor Training

An online training for supervisors of young agricultural workers was developed using a TWH approach and several theoretical perspectives to increase its likelihood of efficacy [24]. The theories included SEM [4], Social Learning Theory [25], and the Extended Parallel Process Model [26,27,28,29,30]. SEM addresses levels of influence (i.e., supervisors and policy). Social Learning Theory points to the importance of modeling safety behaviors (e.g., supervisors also engaging in behaviors to reduce injury). The EPPM emphasizes the importance of addressing both threat (i.e., the severity and susceptibility of young workers to injury) and efficacy (i.e., skills to perform behaviors to protect young workers and that the recommended solutions will address the problem).

The supervisor training begins by describing the threat to young workers in agriculture. This includes developmental differences (both physical and cognitive), the impact of inexperience, risky behaviors that tend emerge in young adulthood (e.g., substance use), and the difficulties of balancing work, school, and home which impact stress and fatigue. Efficacy was addressed by providing and demonstrating specific skills supervisors can use to reduce threat. These include health communication skills to ensure the delivery of effective occupational safety and health training [31], with a particular focus on using the teach-back method and open-ended questions [32]. These skills are particularly important among supervisors of this population. Young workers may be reluctant to speak up if they are uncertain of the task requirements or if they have a concern. In addition, the training addresses the need for ongoing supervision, and not just when the worker is hired, how to communicate and enforce policies, and the importance of role modeling.

### 2.3. Instruments

Questionnaires were used at pre-test, immediate post-test, and at a three-month follow-up. The pre-test questionnaire collected information on demographics, workplace characteristics, beliefs about protecting young workers (e.g., “I know what age-appropriate assignments are for the workers I supervise”), and supervisors’ communication behaviors (e.g., talking with young workers safety and health issues). A 25-item knowledge pre-test was administered immediately prior to beginning training. Upon completion of each module within the training, changes in knowledge were assessed (immediate post-test). Attitude change was assessed after all training modules were completed. Three months after completing the training, participants were invited to complete a follow-up questionnaire (3-month post-test). This questionnaire included workplace characteristics, knowledge, beliefs, supervisors’ communication behaviors, and open-ended questions on training impact. All questionnaires were pilot tested during the development of the training [24].

### 2.4. Procedures

A pre-post/post design was used to evaluate training efficacy. All study procedures occurred online. Prospective participants were directed to the project website with a link to study materials. Following online consent, participants completed a pre-test questionnaire lasting approximately 15 min. The pre-test questionnaire collected information on demographics, workplace characteristics, beliefs about protecting young workers (e.g., “I know what age-appropriate assignments are for the workers I supervise”), and supervisors’ communication behaviors (e.g., talking with young workers about safety and health issues). Supervisor beliefs were assessed using a 5-type Likert scale ranging from “strongly disagree” to “strongly agree.” Participants were then directed to the training module, where they completed a 25-item knowledge pre-test prior to beginning the training. The training took approximately 45 min to complete and included knowledge checks throughout. The knowledge checks were used to assess post-training knowledge (immediate post-test). Next, participants completed an immediate post-test questionnaire. Participants received $50 for completing the pre-test questionnaire, training, and immediate post-test questionnaire. Three months after completing the training, participants were invited to complete a follow-up questionnaire, approximately 20 min in length. This questionnaire included workplace characteristics, knowledge, beliefs, supervisors’ communication behaviors, and open-ended questions describing how they applied the training. Participants received an additional $50 for completing the 3-month post-test questionnaire. A list of all measures can be found in Table 1. Up to three email reminders were sent to participants at each step of the study. All study procedures were reviewed and approved by the Institutional Review Board at the University of Iowa.

### 2.5. Statistical Methods

Descriptive statistics were calculated for all measures to characterize demographic and occupational items. McNemar’s test was used to examine behavior changes (i.e., frequency of talking to young workers about safety and risk) at baseline and 3-month follow-up. Linear mixed effects models were used to examine the means of variables at different time points regarding changes in beliefs. Data analysis was completed with SAS 9.4 (SAS Institute, Cary, NC, USA). An alpha of 0.05 was used for testing significance.

## 3. Results

The analyses that follow include participants that completed all parts of the study.

### 3.1. Description of Respondents and Response Rates

**Figure 1:**
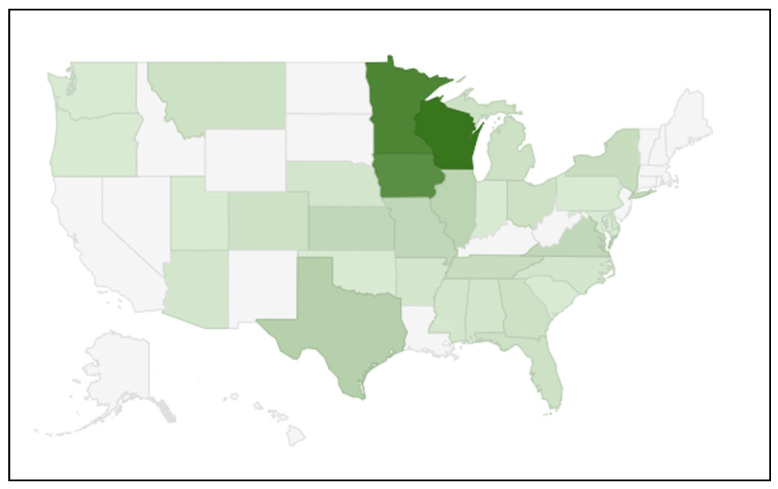
U.S. States with training participants, *n* = 182. States with no shading (white) had no participants. Darker shading represents a greater number of participants. Participants from Canada and U.S. territories are not included in the figure.

Three-hundred-ninety-two respondents consented to participate in the study. Of these, 319 completed the pre-test questionnaire, 236 completed the training, 204 completed the immediate post-test questionnaire, and 185 completed the 3-month follow-up questionnaire. One-hundred-eighty-two participants completed all parts of the study. At each successive step, absolute response rates were 81.0%, 74.0%, 86.4%, and 90.7%, respectively. Cumulative response rates were 81.0%, 59.9%, 51.8%, and 47.0%, respectively. Participants represented 31 states, 2 U.S. territories (District of Columbia and Puerto Rico), and 3 provinces in Canada (Alberta, Manitoba, and Saskatchewan) (Figure 1).

### 3.2. Demographic and Workplace Characteristics

The majority of participants were female and agricultural education teachers. On average, participants had worked in agriculture nearly 18 years. Two-thirds had at least five years of experience supervising young agricultural workers (Table 2). At pre-test, participants reported supervising a median of 22.5 workers between the ages of 14–20 in the previous six months. Fifty percent of participants had supervised between 5 and 75 workers in the previous 6 months. There were no significant differences in demographic characteristics among respondents by occupation (ag teachers versus all other occupations) or study completion (those who completed all parts of the study versus those who dropped out).

### 3.3. Changes in Knowledge

Pre-test knowledge scores were high ($\bar{X}$ = 81.7%) and showed significant improvement immediately following the training. However, knowledge scores decreased at the 3-month follow-up (Table 3).

### 3.4. Changes in Beliefs

Supervisors were asked about their beliefs regarding training and other behaviors they can utilize to protect younger workers, including the impact of activities outside of work on workplace safety. Supervisors rated 11 beliefs on a Likert-type scale (1 = strongly disagree to 5 = strongly agree). The teach-back method (*The teach-back method is effective in training workers under the age of 21*) also included the option “I don’t know what the teach-back method is.” Agreement with the belief statement increased for all items from pre-test to immediate post-test and changes were sustained at the 3-month follow-up. Only two beliefs did not show significant changes following the training (Table 4 and Table 5).

### 3.5. Supervisors’ Communication Behavior Changes

We observed an increase in the percentage of supervisors talking to young workers about safety issues at various timepoints: (1) when they start a new task, (2) when they are hired, and, (3) after an injury or near miss between the pre-test and the 3-month post-test (Table 6). Similarly, we observed an increase from pre-test to 3-month post-test in the percentage of supervisors talking to young workers about other factors that increase risk for injury, such as stress or not enough sleep, when they were assigned to a new task or when they were hired. More importantly, we observed more supervisors reporting discussions of safety occurring every day or at least once a week, rather than conversations that are limited to once a month or longer intervals (Table 7). In sum, supervisors are talking about safety and health in more contexts after completing the training (Table 8).

### 3.6. Application of the Training: Qualitative Findings

At the 3-month post-test, participants were presented with optional open-ended questions asking how the training helped them and to share a story about a young worker they supervised. Ninety-five percent (*n* = 172) of participants provided comments; of these, 91% (*n* = 156) indicated they were more prepared to effectively supervise young workers after taking the training. Of the supervisory practices addressed in the training, teach-back was the most popular, with 23% (*n* = 40) of participants referencing it in their comment. One participant shared the following story of how they used the teach-back method to ascertain a young worker’s understanding of a task:

1. “I had a worker who seemed to know what they were doing. Then when I asked them to talk me through the steps and show me, they didn’t know what to do. They said they were embarrassed to admit they didn’t know. Now they know the proper procedure.”

Another participant provided an example of how they changed a supervisory behavior following the training:

2. “We had a young worker who frequently lifted heavy items by hand to load them in the truck. After discussing this risk with him he now uses a cherry picker.”

Nine out of ten participants said they would recommend the training to others. In addition, we also received positive feedback on the training.

3. “Really good information; things I’ll be able to use when working with youth on our farm. We are very aware of what a dangerous line of work we are in!”
4. “I have completed the training and thought the training information was presented well and would like my supervisors to also participate. I also like that there is a Certificate of Training Completion available once the training is complete.”

## 4. Discussion

A theoretically based and TWH approach were used to develop an online training for supervisors (e.g., employers, educators, parents) of young agricultural workers. Adolescents and young adults (<25 years) working in agriculture are at greater risk of injury than youth working in other industries [1,2,3]. Supervisors play an important role in protecting these young workers who may lack workplace experience and who’s bodies and brains are still developing [4,21,22,23]. The training incorporated materials from existing trainings focused on working youth and utilized a TWH approach to address an expanded view of occupational safety that not only addresses injury prevention, but also focuses on health promotion and worker well-being.

A pre-post/post study design was used to evaluate the training among supervisors. One-hundred-eighty-two participants completed all parts of the efficacy trial. Participants included agricultural educators, farmers and producers, supervisors, and safety and health professionals. The overall reaction to the training was positive. Positive changes in knowledge, beliefs, and supervisors’ communication behaviors were observed.

### 4.1. Significant or Key Findings

In this efficacy trial, training intervention produced significant and sustained changes in beliefs and supervisors’ communication behaviors that are likely to impact the occupational health and safety of young, vulnerable, agricultural workers. Although knowledge increased at immediate post-test, it was not sustained at 3-month follow-up. At pre-test, knowledge was high, which may reflect the years of supervisory experience of our participants. These findings indicate that immediately after completing the training, supervisors had a greater understanding of the risks to young workers. At 3-month follow-up they were more likely to engage in behaviors to protect the safety and health of young workers than they were prior to taking the training. Positive changes in when, how, and under what circumstances supervisors talk about safety and health with their young workers occurred. Establishing patterns of protective behaviors in the workplace (including family farms) can have lifelong impact, particularly among young workers. While additional research is needed, these findings indicate that an online training targeting supervisors is a promising approach to changing safety and health behaviors in agricultural settings.

### 4.2. Dissemination

Dissemination activities included promoting the training through multiple channels to a broad audience. Materials were disseminated through the Agricultural, Forestry, and Fishing Centers of Excellence funded by the National Institute for Occupational Safety and Health (NIOSH AgFF) as well as through programs addressing agricultural safety and health, employers, commodity groups, extension, and agricultural educators. They are listed as a resource as part of the Cultivate Safety materials (https://cultivatesafety.org, accessed on 31 July 2021). They are also promoted through the Great Plains Center for Agricultural Health as part of other online modules addressing agricultural safety health and integrated into the curriculum of Agricultural Safety and Health courses at the University of Iowa. These materials can be used by anyone who assigns tasks in the workplace, provides training, develops policies to address safety, provides supervision, or has regular and frequent opportunities to communicate with workers. Although designed for employers, the training may also be relevant to parents who have children working on a family farm as well as high school agricultural teachers, 4-H leaders, and FFA advisers.

In addition to the online training, Supplementary Materials were developed including a classroom presentation, ten videos, and two short “toolbox-talk” activities that emphasize different aspects of the training. All training and materials are available in English and Spanish. We took care to ensure that materials developed during the project are housed in Translation Supporting Structures [33]. Specifically, the use of the Healthier Workforce Center of the Midwest (https://hwc.public-health.uiowa.edu/protecting-young-ag-workers/, accessed on 31 July 2021) and the Institute for Public Health Practice (https://www.training-source.org/category-pilms/agricultural-health, accessed on 31 July 2021) websites to house the intervention and other materials developed will ensure that the evidence-based intervention remains freely accessible to key audiences.

### 4.3. Research Outcomes/Impact

The development of material involved the integration of health promotion, health protection, TWH, and evidence-based communication approaches to provide supervisors the tools to increase the likelihood of impacting worker behavior. Behaviors emphasized in the training (e.g., teach-back, role modeling, asking open-ended questions, and enacting workplace policies) are transferable to many safety and health risks. These behaviors are not limited to specific agricultural operations, but rather fit all workplaces.

The efficacy trial demonstrated changes in knowledge, beliefs, and supervisors’ communication behaviors. These short-term and moderate-term (3 month) impacts are essential to longer-term changes in morbidity and mortality [34,35]. We successfully altered supervisors’ behaviors through the implementation of communication science [31]. In the long-term, these behaviors will lead to organizational changes that will impact the safety and health of vulnerable agricultural workers. Moreover, altering the safety and health risks of adolescents and young adults establish practices that become integrated into the workplace culture and assist in the development of habits to promote safety, health, and well-being.

Study participants included those with many years of experience supervising young workers in agriculture. Interestingly, changes in beliefs and behaviors were observed among these more experienced supervisors. Although designed primarily for employers, the most eager study participants in the efficacy trial were high school agricultural education teachers. This is a population that is ripe for intervention and dissemination as intermediaries. They have access to a large number of youth and can build expectations about workplace practices and build confidence in youth to talk about safety and health. Future research should target this influential population. Promoting safety and health can lead to changes in the workplace that will not only reduce injuries, but also promote health and well-being on and off the farm.

## 5. Conclusions

This project was unique in its comprehensive approach to addressing the combination of both preventing workplace injuries (health protection) and promoting healthy lifestyle behaviors, utilizing a Total Worker Health approach. Moreover, the addition of workplace policies expanded the intervention to address multiple levels of influence in the Social Ecological Model: supervisors and workplaces. The Extended Parallel Process Model was used to address elements in the intervention necessary to change supervisors’ behaviors. An efficacy trial demonstrated training effectiveness, specifically positive increases in knowledge, beliefs, and supervisors’ communication behaviors.

## Figures and Tables

**Table 1:** Table of Measures, number of items, and response categories.

Measure	Pre-Test	Immediate Post-Test	3-Month Post-Test
Demographics and workplace characteristics *13 items; Multiple choice and open-ended*	X		
Knowledge of safety and health promotion *25 items; Multiple choice*	X	X	X
Supervisor beliefs (age-appropriate assignments, modeling, workplace policies, regular training, using teach-back, open-ended questions, outside of work factors impacting safety on the job) *11 items; 5-point Likert scale ranging from 1 (Strongly Disagree) to 5 (Strongly Agree)*	X	X	X
Supervisors’ communication behaviors (talk to young workers about safety issues, i.e., PPE, ATV * safety, and other factors that increase risk of injury, i.e., stress, not enough sleep) *2 items; Select all that apply (when they are hired, when you assign a new task, after an injury or near miss, never, once a year, once a month, once a week, everyday)*	X		X

* All-terrain vehicle

**Table 2:** Demographic and workplace characteristics.

Item	All (*n* = 182)	
	Mean or % (*n*)	Range
Age	35.6	22–71
Gender:		
Female	63.7 (116)	
Male	35.7 (65)	
Other	0.55 (1)	
Years supervising workers under 21:		
≥5	66.5 (121)	
<5	31.9 (58)	
Do not supervise under 21	1.65 (3)	
Occupation *:		
Agricultural Education Teacher	69.2 (126)	
Farmer/Producer	20.3 (37)	
Supervisor	12.6 (23)	
Safety and Health Professional	8.2 (15)	
Other	14.3 (26)	
Years working in agriculture	17.8	0–60
Commodity:		
I do not work on a farm	45.1 (82)	
Livestock (i.e., pork, poultry)	29.1 (53)	
Row crop (i.e., corn, soybeans)	11.5 (21)	
Other	14.3 (26)	

* Percentages do not equal 100 as respondents could select multiple categories.

**Table 3:** Average percent correct on knowledge questions.

Time	Average Percent Correct (SD)
Pre-test	81.7 (9.57)
Immediate Post-test	89.3 (6.90) *
3-Month Post-test	83.0 (9.79)

* Significant increase at immediate post-test.

**Table 4:** Changes in supervisor beliefs regarding training and other behaviors they can utilize to protect younger workers and their importance over time.

Time	Strongly Disagree	Somewhat Disagree	Neither Agree nor Disagree	Somewhat Agree	Strongly Agree	Pre- to Post-Test Pre- to 3-Month
	% (*n*)	% (*n*)	% (*n*)	% (*n*)	% (*n*)	*p*-Value
I know what age-appropriate assignments are for the workers I supervise.						
Pre-test	1.1 (2)	4.4 (8)	5.5 (10)	42.3 (77)	46.7 (85)	
Post-test	0.0 (0)	2.2 (4)	0.0 (0)	25.3 (46)	72.5 (132)	<0.0001
3-month	0.6 (1)	0.6 (1)	2.8 (5)	34.6 (63)	61.5 (112)	<0.0001
If supervisors and co-workers do safe things, workers under the age of 21 will do safe things.						
Pre-test	1.6 (3)	4.4 (8)	7.1 (13)	44.0 (80)	42.9 (78)	
Post-test	0.0 (0)	1.1 (2)	1.6 (3)	28.6 (52)	68.7 (125)	<0.0001
3-month	0.0 (0)	1.1 (2)	3.8 (7)	39.6 (72)	55.5 (101)	<0.0001
All workers need to know the workplace policies and what will happen if they don’t follow the rules.						
Pre-test	1.6 (3)	1.6 (3)	2.2 (4)	9.3 (17)	85.2 (155)	
Post-test	0.0 (0)	0.6 (1)	0.0 (0)	9.9 (18)	89.5 (162)	0.0230
3-month	0.5 (1)	1.1 (2)	0.0 (0)	11.0 (20)	87.4 (159)	0.1965
Workers under the age of 21 need regular training which should be frequently repeated.						
Pre-test	1.1 (2)	2.2 (4)	6.6 (12)	25.3 (46)	64.8 (118)	
Post-test	0.0 (0)	0.6 (1)	0.0 (0)	9.9 (18)	89.5 (162)	<0.0001
3-month	0.5 (1)	0.5 (1)	2.7 (5)	21.4 (39)	74.7 (136)	0.0032
Questions about a task that end with a “yes” or “no” answer will not tell me if a worker under the age of 21 has thought about safety.						
Pre-test	1.6 (3)	9.3 (17)	17.0 (31)	45.1 (82)	26.9 (49)	
Post-test	4.4 (8)	1.1 (2)	1.1 (2)	16.6 (30)	76.8 (139)	<0.0001
3-month	4.4 (8)	3.3 (6)	5.5 (10)	23.1 (42)	63.7 (116)	<0.0001
Training workers about safety is an ongoing process.						
Pre-test	1.1 (2)	0.5 (1)	0.5 (1)	11.0 (20)	86.8 (158)	
Post-test	0.6 (1)	0.0 (0)	0.6 (1)	5.0 (9)	93.9 (170)	0.0518
3-month	0.5 (1)	0.0 (0)	0.0 (0)	9.3 (17)	90.1 (164)	0.2626
Safety is more important than working quickly.						
Pre-test	1.1 (2)	0.0 (0)	2.2 (4)	9.3 (17)	87.4 (159)	
Post-test	0.5 (1)	0.0 (0)	0.5 (1)	6.6 (12)	92.3 (168)	0.0672
3-month	0.0 (0)	0.0 (0)	0.5 (1)	6.6 (12)	92.9 (169)	0.0137
Talking with workers under the age of 21 frequently can help prevent injuries.						
Pre-test	1.6 (3)	0.5 (1)	3.8 (7)	37.4 (68)	56.6 (103)	
Post-test	0.6 (1)	0.0 (0)	1.7 (3)	15.5 (28)	82.3 (149)	<0.0001
3-month	0.0 (0)	0.5 (1)	1.1 (2)	25.8 (47)	72.5 (132)	<0.0001
Being approachable can help prevent injuries.						
Pre-test	1.1 (2)	0.0 (0)	2.7 (5)	20.9 (38)	75.3 (137)	
Post-test	0.6 (1)	0.0 (0)	0.6 (1)	9.4 (17)	89.5 (162)	0.0003
3-month	0.0 (0)	0.0 (0)	1.1 (2)	11.0 (2)	87.9 (160)	0.0005
Distractions at home, such as stress about friends or homework, can increase the risk of injury at work for workers under the age of 21.						
Pre-test	1.1 (2)	0.0 (0)	2.7 (5)	28.6 (52)	67.6 (123)	
Post-test	0.5 (1)	0.0 (0)	0.0 (0)	7.7 (14)	91.8 (167)	<0.0001
3-month	0.0 (0)	0.5 (1)	0.5 (1)	13.2 (24)	85.7 (156)	<0.0001

**Table 5:** Changes in supervisor beliefs about teach-back method effectiveness and importance over time.

Time	Do Not Know Teachback Method	Strongly Disagree	Somewhat Disagree	Neither Agree nor Disagree	Somewhat Agree	Strongly Agree	Pre- to Post-Test Pre- to 3-Month
	% (*n*)	% (*n*)	% (*n*)	% (*n*)	% (*n*)	% (*n*)	*p*-Value
The teach-back method is effective in training workers under the age of 21.							
Pre-test	49.5 (90)	0.6 (1)	0.6 (1)	5.5 (10)	17.0 (31)	26.9 (49)	
Post-test	4.4 (8)	4.7 (3)	0.0 (0)	0.0 (0)	11.5 (21)	82.4 (150)	<0.0001
3-month	6.0 (11)	0.0 (0)	0.0 (0)	1.1 (2)	14.3 (26)	78.6 (143)	0.0004

**Table 6:** Changes in supervisors’ frequency of whether they talk to young workers about safety and health issues (yes/no) that impact their risk of injury (*n* = 182).

Item	Pre-Test	3-Month Post-Test	Pre-Test to 3-Month
	% (*n*)	% (*n*)	*p*-Value
Talk to young workers about safety issues that increase risk of injury *			
When they are hired	48.3 (88)	55.6 (101)	0.5903
When you assign a new task	60.4 (110)	66.5 (121)	0.0002
After an injury or near miss	24.2 (44)	28.0 (51)	<0.001
Do not talk at any of these times	26.9 (49)	23.1 (42)	<0.001
Talk to young workers about health issues that increase risk of injury *			
When they are hired	45.2 (84)	51.7 (94)	0.760
When you assign a new task	40.1 (73)	47.3 (86)	0.077
After an injury or near miss	25.3 (46)	26.4 (48)	<0.0001
Do not talk at any of these times	35.7 (65)	30.8 (56)	<0.0001

* Percentages do not equal 100 as respondents could select multiple categories.

**Table 7:** Changes in supervisors’ frequency of how often they talk to young workers about safety and health issues that impact their risk of injury (*n* = 182).

Item	Pre-Test	3-Month Post-Test	
	% (*n*)	% (*n*)	*p*-Value
Talk to young workers about safety issues that increase risk of injury			
No answer	40.7 (74)	31.3 (57)	0.0003
Never	0.55 (1)	1.65 (3)	<0.0001
Once a year	13.7 (25)	8.24 (15)	<0.0001
Once a month	12.1 (22)	8.24 (15)	<0.0001
Once a week	13.7 (25)	24.2 (44)	<0.0001
Every day	19.2 (35)	26.4 (48)	<0.0001
Talk to young workers about health issues that increase risk of injury			
No answer	41.2 (75)	34.1 (62)	0.0013
Never	0.0 (0)	0.0 (0)	--
Once a year	14.3 (26)	6.59 (12)	<0.0001
Once a month	15.9 (29)	14.8 (27)	<0.0001
Once a week	14.8 (27)	26.9 (49)	<0.0001
Every day	13.7 (25)	17.6 (32)	<0.0001

**Table 8:** Changes in the number of contexts that supervisors talk about safety and health (i.e., at hire, when assigning tasks, and/or following a near miss/injury) before and after completing the training (*n* = 182) *.

Item	Pre-Test	3-Month Post-Test	
	% (*n*)	% (*n*)	*p*-Value
Number of contexts in which supervisors talk to young workers about safety issues that increase risk of injury			
0	26.9 (49)	23.1 (42)	<0.0001
1	28.0 (51)	23.6 (43)	<0.0001
2	30.2 (55)	33.5 (61)	<0.0001
3	14.8 (27)	19.8 (36)	<0.0001
Number of contexts in which supervisors talk to young workers about health issues that increase risk of injury			
0	35.7 (65)	30.8 (56)	<0.0001
1	28.6 (52)	25.8 (47)	<0.0001
2	24.2 (44)	30.8 (56)	<0.0001
3	11.5 (21)	12.6 (23)	<0.0001

* 0 = supervisors do not talk about safety and health in any context and 3 = talk about safety and health in all contexts.

## Data Availability

The data presented in this study are available on request from the corresponding author.

## Supplementary Materials

The following are available online at https://hwc.public-health.uiowa.edu/protecting-young-ag-workers/. Online training: English/Español. Classroom training: English/Español. YouTube videos: English/Español. Let’s Talk activities: English/Español.
